# Clinicopathological significance of deoxycytidine kinase expression in esophageal squamous cell carcinoma

**DOI:** 10.3892/mco.2013.114

**Published:** 2013-05-09

**Authors:** YUTAKA SHIMADA, TOMOYUKI OKUMURA, SHINICHI SEKINE, MAKOTO MORIYAMA, SHOZO HOJO, KOSHI MATSUI, SHIGEAKI SAWADA, TAKUYA NAGATA, JUNYA FUKUOKA, KAZUHIRO TSUKADA

**Affiliations:** 1 Departments of Surgery and Science, University of Toyama, Toyama, Japan; 2 Surgical Pathology, Graduate School of Medicine and Pharmaceutical Sciences for Research, University of Toyama, Toyama, Japan

**Keywords:** esophageal squamous cell carcinoma, deoxycytidine kinase, gemcitabine, prognostic factor

## Abstract

Deoxycytidine kinase (dCK) mediates the rate-limiting catabolic step in the activation of gemcitabine. Gemcitabine is a key drug for pancreatic and biliary tract cancer. However, gemcitabine is not widely used for esophageal squamous cell carcinoma (ESCC). In this study, we analyzed the expression of dCK in ESCC and evaluated the possibility of gemcitabine treatment for ESCC. In total, 76 ESCC patients who underwent esophagectomy between 1990 and 2008 were analyzed. dCK expression was analyzed immunohistochemically using tissue microarray and compared to the clinocopathological characteristics of the patients. Results identified 41 patients positive for dCK and 35 patients negative for dCK. A significant association was observed between dCK expression and gender (P=0.01), whereas the remaining factors were not associated with dCK expression. Prognosis of the patients with a high dCK expression was significantly worse than that of the patients with a low dCK expression (P=0.022). Furthermore, dCK expression was an independent prognostic factor regarding cause-specific prognosis (risk ratio, 2.2; P=0.031). In conclustion, the results of the present study suggested that dCK expression is a prognostic factor of the ESCC patients.

## Introduction

Gemcitabine is a key drug for pancreatic and biliary tract cancer. For transportation past the cell membrane, gemcitabine is phosphorylated to its mononucleotide moiety by deoxycytidine kinase (dCK), a rate-limiting enzyme involved in the salvage of deoxyribonucleosides that provides deoxynucleotide triphosphates for replicative and repair DNA synthesis (1). dCK expression is associated with prolonged survival after adjuvant gemcitabine for pancreatic adenocarcinoma (2,3). Previously, we reported that the gallbladder carcinoma cell lines with dCK expression were sensitive to gemcitabine treatment (4).

However, gemcitabine is not widely used for the treatment of esophageal carcinoma. At present, few studies are availabe regarding the use of gemcitabine treatment in esophageal cancer and most of the targets involved adenocarcinoma (5–8). Furthermore, no studies regarding dCK expression of esophageal squamous cell carcinoma (ESCC) patients have been reported thus far. In the present study, dCK expression in ESCC was analyzed and compared to the clinocopathological characteristics of the patients.

## Patients and methods

### Patient characteristics and tissue microarray

**Figure 1.**
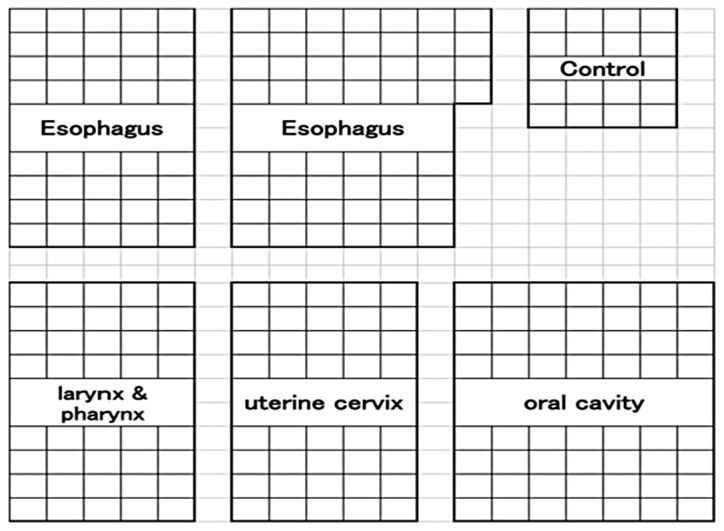
Design of the squamous cell carcinoma (SCC) Ca284 TMA. The layout shows the location of the esophageal, laryngeal and pharyngeal, uterine/cervical and oral cancer.

A squamous cell carcinoma tissue microarray was produced using ESCC, laryngeal and pharyngeal SCC, uterine/cervical SCC and oral SCC. Tumor areas were selected with matched hematoxylin and eosin (H&E)-stained slides and marked directly on the donor block. The cylindrical tissue sample was cored (diameter, 0.6 mm) from the selected region in the donor block and extruded directly into the recipient block. Sections (4 *μ*m) were cut with a microtome and transferred to glass slides (Fisherbrand, Superfrost Plus, Thermo Fisher Scientific, Waltham, MA, USA) (9,10). In total, 114 ESCC patients who underwent esophagectomies between 1990 and 2008 were included in this array (Fig. 1).

### Immunohistochemistry

A rabbit anti-dCK polyclonal antibody (LS-B1825, Lifespan Biosciences, Seattle, WA, USA) was used at a dilution of 1:200. Glass slides with the primary antibodies were incubated on an optimized titer and diluted using universal blocking reagent (BioGenex, Fremont, CA, USA) for 60 min. After washing three times with phosphate-buffered saline (PBS), the slides were incubated for 30 min with biotinylated secondary antibodies (Vector Laboratories, Bulingame, CA, USA) diluted to 1:250 by universal blocking reagent. The slides were then washed three times in PBS and incubated for 45 min with the avidin-biotin complex method reagent (Vectastain Elite ABC kit; Vector Laboratories). The reaction products were rinsed twice with PBS, placed in 0.05 M Tris-HCl buffer (pH 7.5) for 5 min and developed with liquid 3,3’-diaminobenzidine (Dako, Glostrup, Denmark) for 3 min. Thereafter, the slides were washed twice with distilled water, lightly counterstained with Mayer’s hematoxylin, dehydrated, cleared and mounted with a resinous mounting medium. Procedures were carried out at room temperature (10).

### Immunohistochemical analysis

Two investigators analyzed the expression of each gene independently and scored the intensity of expression as 0, no expression; 1, weak expression; 2, moderate expression or 3, strong expression. They also scored the distribution of expression as 0, none; 1, 1–50% of tumor cells; or 2, 50–100% of tumor cells. On the basis of the total score, each patient was then classified into the low expression group (lower group: total score of 0–3) or high expression group (higher/upper group: total score of 4–5).

### Statistical analysis

The Chi-square test, Fisher’s exact test and Student’s t-test were used to compare clinicopathological data. The overall survival (OS) rate and the cause-specific survival (CSS) rate after surgery were calculated for each group by the Kaplan-Maier method and differences were assessed by the log-rank test. P<0.05 was considered to indicate a statistically significanct difference. Analyses were performed using JMP 9.0 software (SAS Institute Inc., Cary, NC, USA).

## Results

### Patient characteristics

Out of 114 spots of ESCC, 84 spots were diagnosed as appropriate for the evaluation. Of these, 8 patients received preoperative chemotherapy and were not eligible. The remaining 76 ESCC patients (67 male and 9 female patients; average age, 64.2 years old) were analyzed in this study. These patients underwent R0 resections. TNM stage (version 6) of the patients was as follows: stage 1, 10; stage 2a, 15; stage 2b, 10; stage 3, 36 and stage 4, 7. All M1 were distant lymph node metastasis with no organ metastasis and were surgically removed. Forty-one patients received postoperative cisplatin based chemotherapy.

### dCK expression in ESCC patients and its prognostic impact

**Figure 2.**
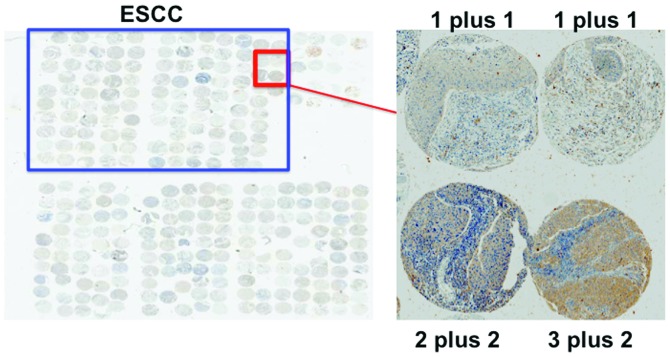
Deoxycytidine kinase (dCK) expression in the squamous cell carcinoma (SCC) tissue array. Out of 114 spots, 84 spots were considered evaluable for immunohistochemical analysis. The samples in the upper section of the inset (red) were diagnosed with a negative expression of dCK and those in the lower section of the window were diagnosed with a positive expression of dCK. ESCC, esophageal squamous cell carcinoma.

**Figure 3.**
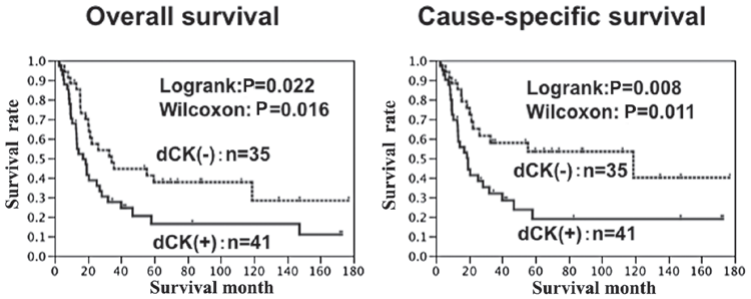
Survival curves of the esophageal squamous cell carcinoma (ESCC) patients. The left panel shows overall survival and the right panel shows cause-specific survival. The prognosis of the patients with a high expression of deoxycytidine kinase (dCK) was significantly worse than that of the patients with a low expression of dCK.

Forty-one patients were positive for dCK and 35 patients were negative for dCK (Fig. 2). There was a significant association between dCK expression and gender (P=0.01). However, there was only a minor association between dCK expression and depth of tumor, lymph node metastasis or pathological stage (P=0.19, P=0.14 and 0.10 respectively) (Table I). The prognosis of the patients with a high expression of dCK was significantly worse than that of the patients with a low expression of dCK (P=0.022) (Fig. 3). Although dCK expression was not an independent prognostic factor regarding overall survival, dCK expression was an independent prognostic factor regarding cause-specific prognosis (risk ratio 2.2, P=0.031) (Tables II and III).

## Discussion

Results of the present study suggest an association of gender and dCK expression. Sebastiani *et al* also reported that dCK expression in male patients was higher than that in female patients (11). Thus, dCK expression may be associated with gender, smoking or alcohol.

Gemcitabine is a key drug for pancreatic and biliary tract cancer. However, gemcitabine is not widely used for the treatment of esophageal carcinoma, and a limited number of studies have focused on gemcitabine treatment in esophageal cancer (5–8). Findings of those studies suggest that gemcitabine alone or as gemcitabine-cisplatin combination were tolerable. However, gemcitabine with irinotecan or gemcitabine with paclitaxel was highly toxic (12,13). Furthermore, there were no additional survival benefits. Thus, gemcitabine was not a standard treatment regimen for esophageal cancer. However, most of their targets involved adenocarcinoma. (Table IV).

By contrast, a phase I study for solid malignancy revealed that 4 cases with response to treatment were ESCC or transitional cell carcinoma (14). Furthermore, Millar *et al* (15) suggested that the response rate appears to be greater in patients with squamous cell carcinoma compared to those with adenocarcinoma. Huang *et al* (16) revealed that a cisplatin-gemcitabine regimen was manageable and had significant efficacy in patients with ESCC as improved survival time was observed. Findings of the abovementioned reports suggested that gemcitabine may be more effective against ESCC as compared to esophageal adenocarcinoma (Table IV). Thus, although our results suggest that ESCC with dCK-positive patients have a worse prognosis, gemcitabine treatment is expected to improve the prognosis of ESCC patients. However, to confirm the usefulness of dCK for gemcitabine treatment in ESCC, prospective clinical trials should be performed based on dCK expression.

In conclusion, results of the present study suggest that dCK expression is a prognostic factor for ESCC patients. Therefore, dCK-positive ESCC patients may be optimal targets for gemcitabine treatment.

## Figures and Tables

**Table I.** Patient characteristics.

Variables	dCK (+)	dCK (−)	P-value
Age, years	62.5±11.0	66.2±8.0	0.10
Gender			0.01
Male	40	27	
Female	1	8	
Depth of tumor			0.19
T1	5	11	
T2	7	6	
T3	23	13	
T4	6	5	
Lymph node metastasis			0.14
N0	11	15	
N1	30	20	
Distant metastasis^a^			0.44
M0	36	33	
M1	5	2	
TNM stage			0.10
1	3	7	
2a	7	7	
2b	3	7	
3	23	12	
4	5	2	
Histological type			0.55
Well-mod	32	30	
Por	9	5	
Adjuvant chemotharapy			0.38
No	17	18	
Yes	24	17	

a No organ metastasis. dCK, deoxycytidine kinase. Well, well-differentitated; mod, moderately differentiated; por, poorly differentiated.

**Table II.** Correlation between patient characteristics and the overall prognosis in ESCC, assessed by univariate and multivariate analyses.

Variables	Univariate analysis	Multivariate analysis		
	P-value	Risk ratio	95% CI	P-value
Age (>65 years)	0.717	1.46	0.75–2.86	0.262
Gender (male)	0.160	1.04	0.37–3.42	0.941
T (>2)	0.001	1.65	0.84–3.42	0.151
N (positive)	<0.001	2.23	1.03–5.16	0.041
M (positive)	0.006	2.25	0.85–5.34	0.098
Histological type (por)	0.263	0.44	0.18–0.98	0.044
Adjuvant chemotherapy (yes)	0.347	1.55	0.81–2.99	0.186
dCK (positive)	0.041	1.83	0.96–3.59	0.065

ESCC, esophageal squamous cell carcinoma; T, tumor; N, node; M, metastasis; CI, confidence interval; por, poorly differentiated; dCK, deoxycytidine kinase.

**Table III.** Correlation between patient characteristics and cause-specific prognosis in ESCC. Univariate and multivariate analyses.

Variables	Univariate analysis	Multivariate analysis		
	P-value	Risk ratio	95% CI	P-value
Age (>65 years)	0.911	1.53	0.73–3.25	0.261
Gender (male)	0.199	0.88	0.27–3.41	0.840
T (>2)	<0.001	2.27	1.02–5.43	0.044
N (positive)	0.002	1.66	0.70–4.22	0.254
M (positive)	0.001	2.69	0.99–6.74	0.053
Histological type (por)	0.276	0.42	0.15–1.01	0.053
Adjuvant chemotherapy (yes)	0.016	1.99	0.96–4.17	0.064
dCK (positive)	0.008	2.34	1.12–5.10	0.022

ESCC, esophageal squamous cell carcinoma; T, tumor; N, node; M, metastasis; CI, confidence interval; por, poorly differentiated; dCK, deoxycytidine kinase.

**Table IV.** Clinical studies for gemcitabine in esophageal cancer.

Author (Refs.)	Study drug	No. of patients ^a^	Histology			Med OS (M)	CR	PR	RR (%)	Cytotoxity (%)
			SCC	ADC	Other					
Sandler *et al* (5)	Gem	21 (17)	6	14	1	5	0	0	0.0	Grade 3–4 anemia (10.5) Granulocytopenia (21)
Urba *et al* (6)	Gem+CDDP	64 (64)	10	52	2	7.3	-	-	-	Neutropenia (31)
Kroep *et al* (7)	Gem+CDDP	36 (34)	12	24	0	9.8	2	12	41.0	Neutropenia (83) Thrombocytopenia (67)
Millar *et al* (15)	Gem+CDDP	42 (32)	14	28	0	11	3	16	45.0 (SCC>ADC, 71 vs. 33 P<0.04)	Neutropenia (37)
Morgan-Meadows *et al* (8)	Gem+5-FU, LV	35	3	32	0	9.8 (1 year; 37.1%)	1	10	31.4	Neutropenia (58)
Wiliamson *et al* (12)	Gem+IRI	57	-	-	-	6.3	-	-	-	4 TRD, neutropenia (35) Thrombocytopenia (16)
Lowy *et al* (13)	Gem+PTX + (FP+radiation)	29	3	26	0	3 years; 36%	4	11	52.0	Increase in postoperative complications
Huang *et al* (16)	Gem+CDDP	38	38	0	0	10 (1 year 36.8%)	2	14	42.1	Leucopenia (44.7)

a Evaluable patients. SCC, squamous cell carcinoma; ADC, adenocarcinoma; Med OS (M), median overall survival (months); CR, complete response; PR, partial response; RR, response rate; TRD, treatment-related death; Gem, gemcitabine; CDDP, cisplatin; 5-FU, 5-fluorouracil; LV, leucovorin; IRI, irinotecan; PTX, paclitaxel; FP, 5-fluorouracil plus cisplatin.

## References

[1] McDonagh EM, Whirl-Carrillo M, Garten Y, Altman RB, Klein TE (2011). From pharmacogenomic knowledge acquisition to clinical applications: the PharmGKB as a clinical pharmacogenomic biomarker resource. Biomark Med.

[2] Fujita H, Ohuchida K, Mizumoto K (2010). Gene expression levels as predictive markers of outcome in pancreatic cancer after gemcitabine-based adjuvant chemotherapy. Neoplasia.

[3] Maréchal R, Mackey JR, Lai R (2010). Deoxycitidine kinase is associated with prolonged survival after adjuvant gemcitabine for resected pancreatic adenocarcinoma. Cancer.

[4] Sekine S, Shimada Y, Nagata T (2012). Establishment and characterization of a new human gallbladder carcinoma cell line. Anticancer Res.

[5] Sandler AB, Kindler HL, Einhorn LH (2000). Phase II trial of gemcitabine in patients with previously untreated metastatic cancer of the esophagus or gastroesophageal junction. Ann Oncol.

[6] Urba SG, Chansky K, van Veldhuizen PJ (2004). Gemcitabine and cisplatin for patients with metastatic or recurrent esophageal carcinoma: a southwest oncology group study. Invest New Drugs.

[7] Kroep JR, Pinedo HM, Giaccone G, Van Bochove A, Peters GJ, Van Groeningen CJ (2004). Phase II study of cisplatin preceding gemcitabine in patients with advanced oesophageal cancer. Ann Oncol.

[8] Morgan-Meadows S, Mulkerin D, Berlin JD (2005). A phase II trial of gemcitabine 5-fluorouracil and leucovorin in advanced esophageal carcinoma. Oncology.

[9] Fukuoka J, Fujii T, Shih JH (2004). Chromatin remodeling factors and BRM/BRG1 expression as prognostic indicators in non-small cell lung cancer. Clin Cancer Res.

[10] Nagata T, Shimada Y, Sekine S (2012). Prognostic significance of NANOG and KLF4 for breast cancer. Breast Cancer.

[11] Sebastiani V, Ricci F, Rubio-Viqueira B (2006). Immunohistochemical and genetic evaluation of deoxycytidine kinase in pancreatic cancer: relationship to molecular mechanisms of gemcitabine resistance and survival. Clin Cancer Res.

[12] Williamson SK, McCoy SA, Gandara DR (2006). Phase II trial of gemcitabine plus irinotecan in patients with esophageal cancer: a Southwest Oncology Group (SWOG) Trial. Am J Clin Oncol.

[13] Lowy AM, Firdaus I, Roychowdhury D (2006). A phase II study of sequential neoadjuvant gemcitabine and paclitaxel, radiation therapy with cisplatin and 5-fluorouracil and surgery in locally advanced esophageal carcinoma. Am J Clin Oncol.

[14] Fleming DR, Glisson SD, Bhupalam L, Michelson GD, Goldsmith GH, LaRocca RV (2000). Phase I study of paclitaxel and day 1/day8 gemicitabine in patients with solid malignancies. Am J Clin Oncol.

[15] Millar J, Scullin P, Morrison A (2005). Phase II study of gemcitabine and cisplatin in locally advanced/metastatic oesophageal cancer. Br J Cancer.

[16] Huang J, Fan QX, Chen L (2011). Long-term outcomes of gemcitabine and cisplatin in patients with recurrent or metastatic esophageal squamous cell carcinoma: a phase II trial. Chin Med J (Engl).

